# Multilobular tumor of the zygomatic bone in a dog

**Published:** 2014-02-09

**Authors:** L. Leonardi, A. Carrano, L. Stoppini, M. Floris

**Affiliations:** 1 *Dipartimento di Scienze Biopatologiche e Igiene delle Produzioni Animali e Alimentari, Università degli Studi di Perugia, Italy*; 2 *Clinica Euroveterinaria, Via del Colle 12, 06084 Bettona, Perugia, Italy*

**Keywords:** Dog, Multilobular tumor of bone, Zygomatic bone

## Abstract

Multilobular tumor of bone (MTB) (also known as Multilobular Osteochondrosarcoma) is an uncommon bone tumor frequently located on the skull of dogs, rarely on the ribs or pelvis. These neoplasms are slow growing, locally invasive, and have the potential to compress and invade the brain. A 10-year-old mixed breed dog was presented with a history of approximately 4 months of progressive growth of a left zygomatic mass. Radiographic investigation revealed a finely granular or stippled non homogeneous radiopaque mass involving the zygomatic arch. After surgery, grossly the neoplasm consisted of multiple, variably sized, grayish-white to yellow nodules separated by collagenous septa of different thickness. Histologically, the tumor was characterized by the presence of multiple lobules containing osteoid and cartilage, separated by a net of fibrous septae. This neoplastic pattern was consistent with a typical multilobular tumor of bone and based on clinical, radiographical, gross and light microscopic findings the definitive diagnosis was made. While reviewing veterinary literature only few cases of MTB were found in dogs.

## Introduction

Multilobular tumor of bone (MTB) is a slow-growing tumor occurring most often in the skull of dogs (McGavin and Zachary, 2007). It is also called with alternative terms like chondroma rodens, cartilage analogue of fibromatosis, calcifying aponeurotic fibroma, juvenile aponeurotic fibroma, multilobular chondroma or osteoma and multilobular osteochondrosarcoma (Dernell *et al.*, 1998).

In dogs it is uncommon and primarily represents a disease of middle-aged to older animals, occurring most often in medium or large breeds and rarely in giant breeds (Loukopoulos *et al.*, 2003; Jubb *et al.*, 2007). This tumor often recurs locally after surgical excision, and it has been found to metastatize the lungs (McLain *et al.*, 1983; Losco *et al.*, 1984). The tumor is usually present as a firm immovable mass on the surface of skull bones. Direct extension into adjacent structures is common (Hathcock and Newton, 2000; Pakhrin *et al.*, 2006). Depending of the location, the tumor can manifest in various clinical signs in the affected dogs; which include difficulty in mastication, obstruction of sinuses, neurological signs, exophthalmia and disfiguration of the face and head due to the protruding tumor mass (Pakhrin *et al.*, 2006; Psychas *et al.*, 2009).

The literature regarding MTB is limited to a relatively small number of cases reported in dogs (McLain *et al.*, 1983; Losco *et al.*, 1984; Straw *et al.*, 1989; Dernell *et al.*, 1998; Hathcock and Newton, 2000; Loukopoulos *et al.*, 2003; Pakhrin *et al.*, 2006; Webb *et al.*, 2009; Vancil *et al.*, 2012).

### Case Details

**Fig. 1:**
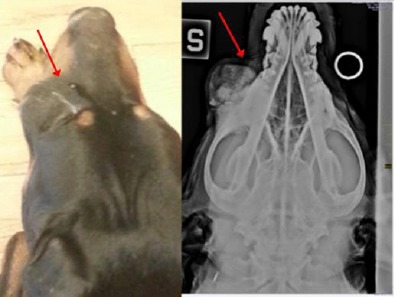
Multilobular Tumor of Bone. Gross (left) and radiologic (right) appearance of the tumor. Radiography shows the dorsoventral projection of the skull and zygomatic area with the mass (arrows).

A 10-year-old mixed medium size breed dog was presented with a 4 months of gradually enlarging swelling in the left zygomatic area of the head. Clinical examination revealed a facial deformation, due to the localization of the mass and mild dyspnea. Neurological examination didn’t reveal any specific dysfunctions. The skin over the mass was tense and no ulcerations were observed. Radiographic examination of the head was performed and revealed the presence of a homogeneous radiodense bony swelling attached to the skull and involved a large area, from the left zygomatic arch to the left nasal cavity (Fig. 1).

The pathognomonic findings of a lytic geographic lesion with expansion and chondroid matrix seen on radiographic were diagnostic for a tumor. Prior to surgery, 3-view thoracic radiographs were performed to assess whether metastatic disease was present but the results were within normal limits. Initial diagnostic tests included a complete blood cell count, serum biochemical profile, prothrombin time (PT) and partially thromboplastin time (PTT).

All results were within reference limits similar to the values on urinalysis on voided urine. The dog was sedated by 5 µgm/Kg bodyweight (BW) of Medetomidine (Domitor 0,2 ml EV) and 0,1 mg/Kg BW of Butorphanol (Dolorex 0,4 ml EV) premedication. The induction was achieved using propofol at 4 mg/Kg BW (17 ml EV) followed by isoflurane for maintenance. The surgery was performed using a lateral access, on the left side of dog’s face. After a careful isolation of anatomic structures and the exposure of the tumor, the mass was removed. Grossly the mass consisted of multiple, variably sized, grayish-white to yellow nodules which occasionally contained whitish, hard areas on cut surface interpreted to be mineral.

The resected mass was fixed in 10% neutral buffered 10% formalin and submitted to the Dipartimento di Scienze Biopatologiche e Igiene delle Produzioni Animali e Alimentari, Università degli Studi di Perugia, Italy.

**Fig. 2:**
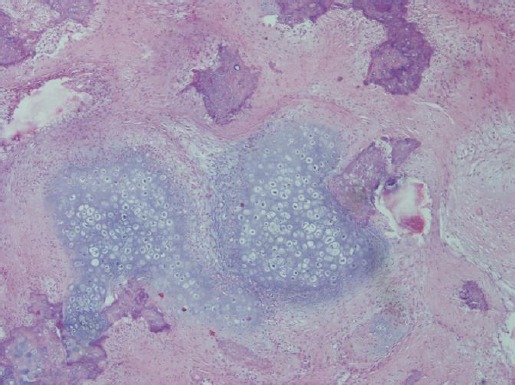
Multilobular Tumor of Bone. Multilobular pattern of irregularly shaped and sized cartilaginous islands separated by fibroblast-like cells. H&E stain, 4x.

Histologically, the tumor was characterized by the presence of multiple lobules of irregularly shaped and sized islands and nests containing well differentiated osteoid and cartilage separated by diffuse anastomosed fibrovascular septa (Fig. 2).

In some areas there were broad poorly differentiated areas of mesenchymal tissue which blended into the lobules and islands of cartilagineous, osseous or osteocartilagineous tissue. Within the islands there were also diffuse areas of ossification and mineralization. The mitotic index of neoplastic cells was always low grade in all the examined samples. According to the characteristics and histological appearance of the tumor a diagnosis of MTB was made.

A radiation therapy was declined by the owner and after 3 months a recurrence was not observed and the dog did not present clinical signs.

## Discussion

MTBs are slow growing, locally invasive and can compress and also invade the brain. They metastatize to the lungs late in the clinical course and the metastases are frequently small and clinically silent (McGavin and Zachary, 2007).

Surgical removal is often difficult because of the location of these tumors and local recurrence occurs in about 50% of the cases (Jubb *et al.*, 2007). Local recurrence is relatively common following surgical excision and is dependent on completeness of surgical resection. Aggressive surgical excision with wide margins remains the treatment of choice and can result in long-term disease remission (Gallegos *et al.*, 2008).

## References

[ref1] Dernell W.S, Straw R.C, Cooper M.F, Powers B.E, LaRue S.M, Whitrow S.J (1998). Multilobular Osteochondrosarcoma in 39 dogs: 1979-1993. J. Am. Anim. Hosp. Assoc.

[ref2] Gallegos J, Schwarz T, McAnulty J.F (2008). Massive midline occipitotemporal resection of the skull for treatment of multilobular osteochondorsarcoma in two dogs. J. Am. Vet. Med. Assoc.

[ref3] Hathcock J.T, Newton J.C (2000). Computed tomographic characteristics of multilobular tumor of bone involving the cranium in 7 dogs and zygomatic arch in 2 dogs. Vet. Radiol. Ultrasound.

[ref4] Jubb K.V.F, Kennedy P.C, Palmer N (2007). Pathology of Domestic Animals: chapter 1 – bone and joints. Volume.

[ref5] Losco P.E, Diters R.W, Walsh K.M (1984). Canine multilobular osteosarcoma of the skull with metastasis. J. Comp. Pathol.

[ref6] Loukopoulos P, Thornton J.R, Robinson W.F (2003). Clinical and pathologic relevance of p53 index in canine osseous tumors. Vet. Pathol.

[ref7] McGavin M.D, Zachary J.F (2007). Pathologic Basis of Veterinary Disease.

[ref8] McLain D.L, Hill J.R, Pulley L.T (1983). Multilobular osteoma and chondroma (chondroma rodens) with pulmonary metastasis in a dog. J. Am. Anim. Hosp. Assoc.

[ref9] Pakhrin B, Bae I.H, Jee H, Kang M.S, Kim D.Y (2006). Multilobular tumor of the mandible in a Pekingese dog. J. Vet. Sci.

[ref10] Psychas V, Loukopoulos P, Polizopoulou Z.S, Sofianidis G (2009). Multilobular tumour of the caudal cranium causing severe cerebral and cerebellar compression in a dog. J. Vet. Sci.

[ref11] Straw R.C, LeCouteur R.A, Powers B.E, Withrow S.J (1989). Multilobular osteochondrosarcoma of the canine skull: 16 cases (1978-1988). J. Am. Vet. Med. Assoc.

[ref12] Vancil J.M, Henry C.J, Milner R.J, McCoig A.M, Lattimer J.C, Villamil J.A, McCaw D.L, Bryan J.N (2012). Use of samarium Sm 153 lexidronam for the treatment of dogs with primary tumors of the skull: 20 cases (1986-2006). J. Am. Vet. Med. Assoc.

[ref13] Webb J.A, Liptak J.M, Hewitt S.A, Vince A.R (2009). Multilobular osteochondrosarcoma of the os penis in a dog. Can. Vet. J.

